# Corrigendum: Interleukin-12 bypasses common gamma-chain signalling in emergency natural killer cell lymphopoiesis

**DOI:** 10.1038/ncomms15185

**Published:** 2017-04-04

**Authors:** Isabel Ohs, Maries van den Broek, Kathrin Nussbaum, Christian Münz, Sebastian J. Arnold, Sergio A. Quezada, Sonia Tugues, Burkhard Becher

Nature Communications 8: Article number: 13708 ; DOI: 10.1038/ncomms13708 (2016); Published: December 16 2016; Updated: march 04 2017

The financial support for this Article was not fully acknowledged. The acknowledgements should have included the following:

The European Community FP7 grant no. 602239 (ATECT).

