# Supplementary material for: Interleukin-12 bypasses common gamma-chain signalling in emergency natural killer cell lymphopoiesis
Source: Nat Commun. 2016 Dec 16;7:13708. doi: 10.1038/ncomms13708 (PMC5172358; doi:10.1038/ncomms13708)
Supplement: Supplementary Information — Supplementary Figures and Supplementary Table. [file ncomms13708-s1.pdf]

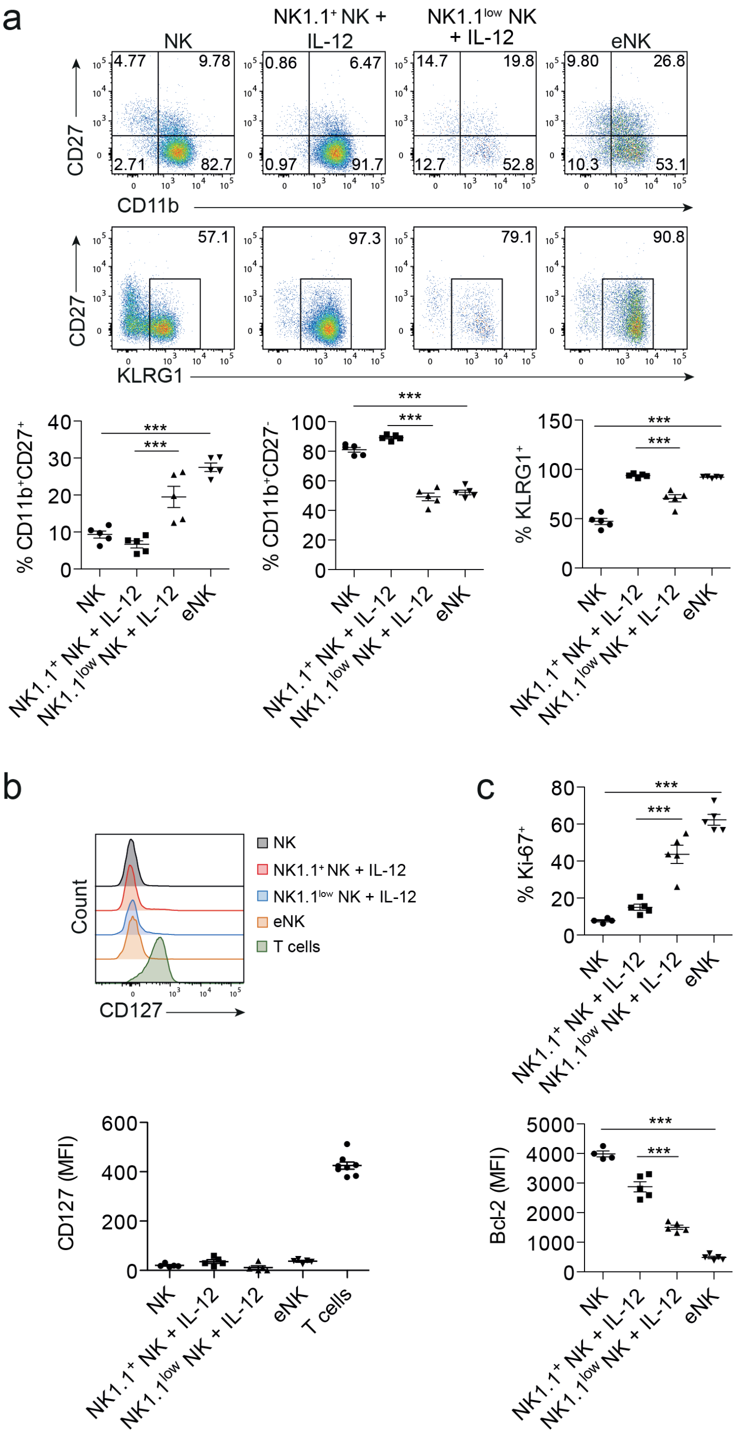

Supplementary Figure 1: (**a**) Expression levels of CD27, CD11b and KLRG1 on the NK1.1^{+} subsets of CD45^{+}CD3^{-}CD122^{+}CD49b^{+} cells isolated from lungs of Ctrl (NK) and IL-12-treated WT mice (NK1.1^{+} NK + IL-12), the NK1.1^{low} subset of CD45^{+}CD3^{-}CD122^{+}CD49b^{+} cells isolated from lungs of IL-12-treated WT mice (NK1.1^{low} NK + IL-12) and eNK cells from *Rag2*$^{-/-}$*Il2rg*$^{-/-}$ mice. Three independent

experiments were performed with at least 3 mice per group. (**b**) Representative histograms and quantification of CD127 expression levels in lung NK, NK1.1^{+} NK + IL-12, NK1.1^{low} NK + IL-12 and eNK cells. Data shown represent two independent experiments. (**c**) Ki-67 and Bcl-2 expression levels were quantified in lung NK, NK1.1^{+} NK + IL-12, NK1.1^{low} NK + IL-12 and eNK cells. Data shown represent three independent experiments (n = 3-5 mice per group per experiment). Data are expressed as mean ± s.e.m. ***$p < 0.001$ as determined by one-way ANOVA with Bonferroni post-test.

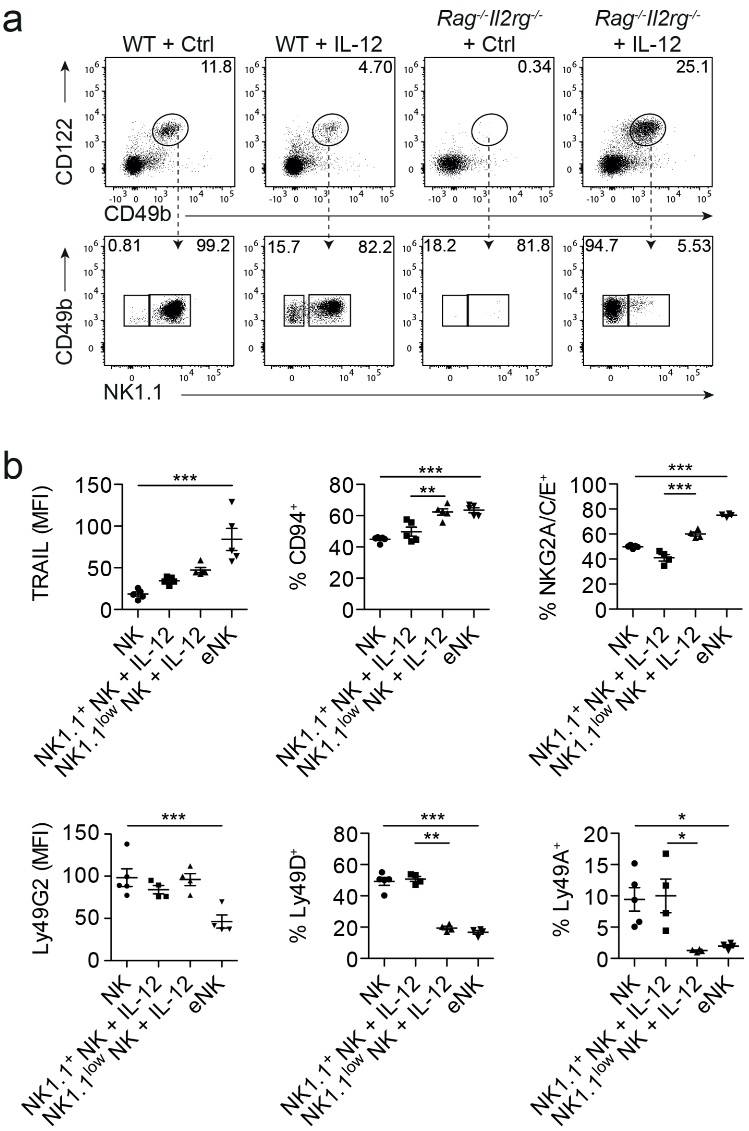

Supplementary Figure 2: (**a**) Representative plots of $CD45^+CD3^-CD122^+CD49b^+$ cells in livers from IL-12 or Ctrl-treated WT or *Rag2*$^{-/-}$*Il2rg*$^{-/-}$ mice. The expression of NK1.1 on $CD45^+CD3^-CD122^+CD49b^+$ cells in livers from IL-12 or Ctrl-treated WT or *Rag2*$^{-/-}$*Il2rg*$^{-/-}$ mice is shown. Data represent two experiments (n ≥ 4 mice per group per experiment). (**b**) Quantification of TRAIL, CD94, NKG2A/C/E, Ly49G2, Ly49D and Ly49A expression on lung NK, $NK1.1^+$ versus $NK1.1^{low}$ NK + IL-12 and eNK cells. Two independent experiments were performed with at least 3-5 mice per group. Data are expressed as mean ± s.e.m. One-way ANOVA was used to determine significance (*$p < 0.05$, **$p < 0.01$, ***$p < 0.001$).

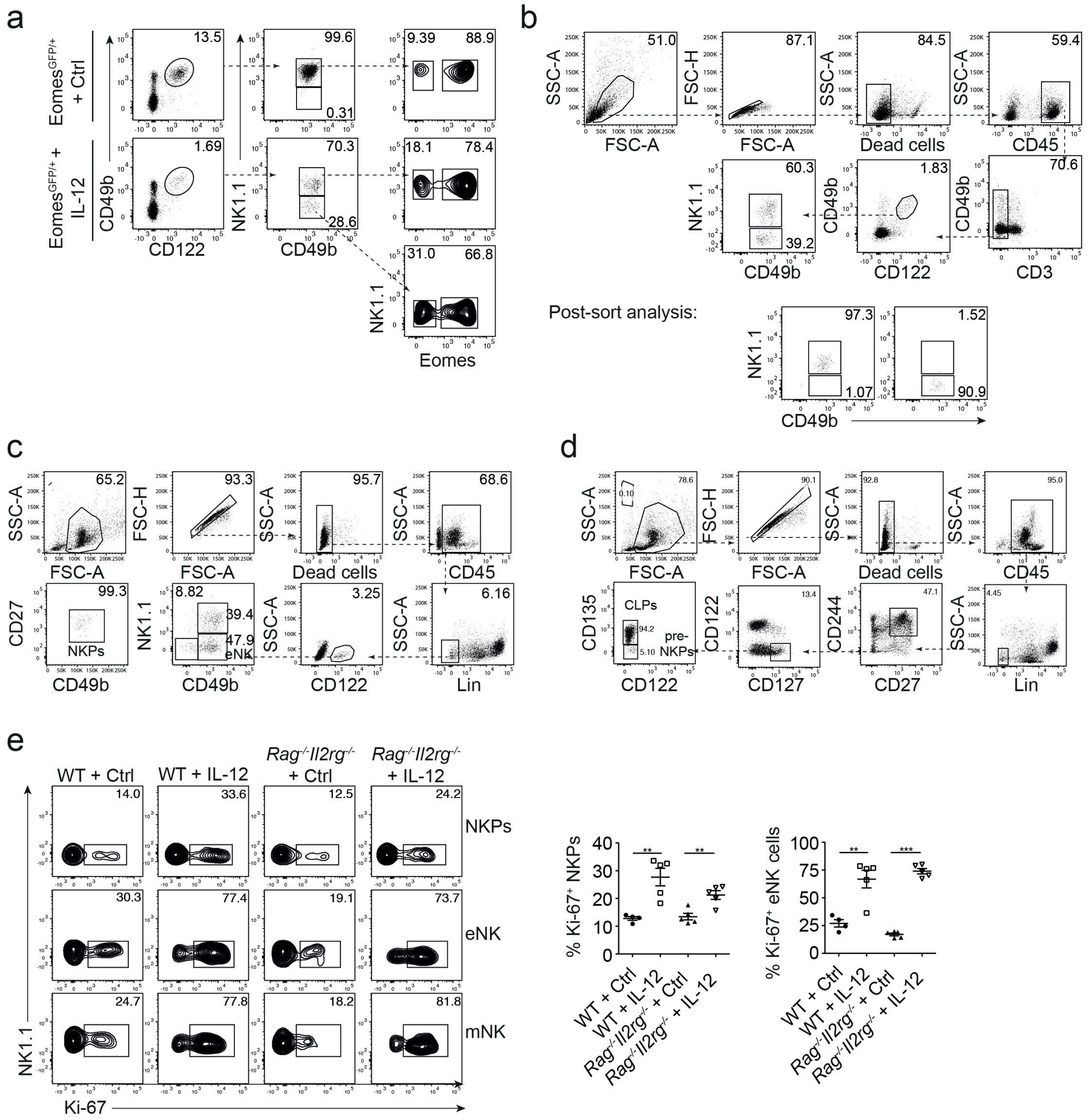

Supplementary Figure 3: (**a**) Eomes expression by CD122$^+$CD49b$^+$NK1.1$^+$ or CD122$^+$CD49b$^+$NK1.1^{low} cells in the BM of Ctrl and IL-12-treated C57BL/6 *Eomes*$^{GFP/+}$ mice. Data are representative of two independent experiments (n = 3 mice per group per experiment). (**b**) Sorting strategy and post-sort analysis of CD45$^+$CD3$^-$ CD122$^+$CD49b$^+$NK1.1$^+$ or CD45$^+$CD3$^-$CD122$^+$CD49b$^+$NK1.1^{low} cells from the spleen of IL-12 treated WT mice. (**c**) Gating strategy for NKPs and eNK cells in the BM is displayed. (**d**) Gating strategy for CLPs and pre-NKPs in the BM is displayed. (**e**) Representative plots and quantification of Ki-67 expression by NKPs, eNK and mNK cells isolated from the BM of WT or *Rag2*$^{-/-}$*Il2rg*$^{-/-}$ mice treated either with Ctrl

or IL-12. Data represent two independently performed experiments with at least four mice per group each. Data are expressed as mean ± s.e.m. (**$p < 0.01$, ***$p < 0.001$, unpaired Student`s t-test with or without Welch`s correction).

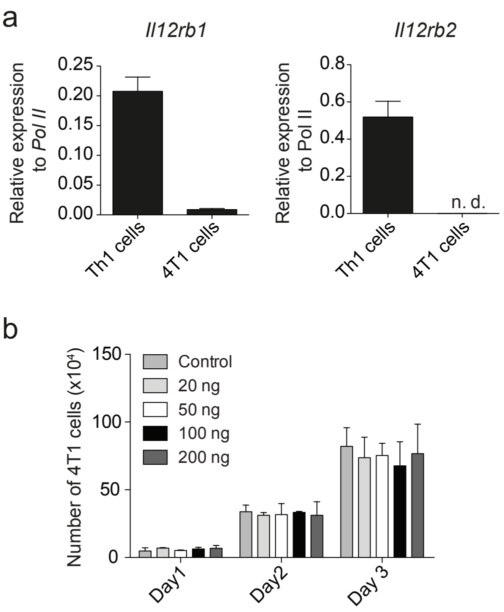

Supplementary Figure 4: (**a**) 4T1 cells were harvested and mRNA expression levels of *Il12rb1* and *Il12rb2* were quantified by qRT-PCR. Representative data of two independent experiments. (**b**) Quantification of 4T1 cells after 1, 2 and 3 days of *in vitro* culture with different concentrations of IL-12. Representative data of two independent experiments. Data are expressed as mean ± s.e.m.

Supplementary Table 1. Expression levels of signature genes in eNK cells compared to NK+IL-12 and NK cells

Cytokines and Cytokine Receptors

Gene symbol	FC: NK + IL-12 / eNK	pValue	NK (1)	NK (2)	NK (3)	NK + IL-12 (1)	NK + IL-12 (2)	NK + IL-12 (3)	eNK (1)	eNK (2)
Il1rl1	-2,761	6.07e-09	95.77	51.32	226.5	422.8	300.2	147.6	1776	2204
Tnf	-2,719	6,50E-05	84.82	23.49	381.5	166.3	136.1	271.3	1742	813.8
Ltb	-2,574	9,67E-11	10640	5738	4080	3515	4079	3941	30320	16170
Il2ra	-1.92	0.00018	9,577	50.45	82.67	460.4	510.8	183.2	948.7	1987
Csf2	-1,725	2.49e-08	206.6	116.6	73.19	2211	2349	3402	9595	8132
Il18r1	-0.9109	0.002915	3219	2874	3086	2031	2054	1783	3558	3873
Il15ra	-0.362	0.4643	333.8	261.8	323.8	527.3	376.9	698.2	956.1	437.5
Il18bp	-0.3459	0.4421	153.2	90.46	57.69	503	584.3	488.2	818.1	535.6
Il16	-0.07169	0.8093	4039	4887	4592	3946	4123	4346	5104	3705
Ifng	-0.04259	0.8996	2816	2472	1851	11570	10120	16810	13900	12780
Ifnar2	-0.03856	0.9182	2318	1315	1330	1021	1185	1018	1211	1023
Tgfbr2	0.2415	0.5334	2256	2373	3692	3548	2844	1541	1953	2567
Ifnar1	0.3271	0.498	759.3	2635	3450	3396	3287	1152	1256	2937
Il10	0.3276	0.2972	1,368	0.8698	14.64	7080	5802	8688	6522	5066
Il12rb1	0.3559	0.3231	640.3	1144	925.7	2995	2431	2326	1462	2603
Il10ra	0.473	0.2035	2639	3426	2867	2561	2472	1430	1522	1618
Il17ra	0.5883	0.1502	1783	6605	8373	7136	8244	4496	2578	6286
Il2rb	0.5901	0.149	17480	27260	32680	49810	44570	46140	22790	39840
Il10rb	0.6108	0.05025	4245	7196	6732	4404	4406	5774	3722	2721
Ifngr1	0.6462	0.06182	12640	16340	22060	15750	16650	23700	12230	11880
Il12rb2	0.7954	0.08026	1447	3885	5682	9608	8818	4041	2399	6283
Il27ra	0.9916	0.001529	1082	2956	2568	3599	4657	4506	2058	2261

Chemokines and Chemokine Receptors

Gene symbol	FC: NK + IL-12 / eNK	pValue	NK (1)	NK (2)	NK (3)	NK + IL-12 (1)	NK + IL-12 (2)	NK + IL-12 (3)	eNK (1)	eNK (2)
Cxcl15	-2,749	3,41E-04	1687	291.4	1470	162.2	79.92	89.13	749.2	754.4
Cxcr6	-2,231	8,34E-09	134.1	104.4	457.2	2664	3486	2801	17900	10540
Cxcr3	-1,907	2,34E-04	1027	1237	582.1	545.5	565.9	709.1	2680	1929
Cxcr4	-1,474	0.05577	648.5	366.2	812	257.6	76.68	414.9	145.2	1237
S1pr1	-1,373	0.0002152	5514	1371	5354	627.7	623.2	852.6	1744	1920
Xcl1	-1,262	7,10E-02	3500	915.1	431.4	1621	1737	2537	4775	4736
Ccl6	-0.8596	0.0477	4522	1161	3268	568.9	504.4	835.8	863.1	1458
Mif	-0.7993	0.06257	1590	527.1	305.7	452.3	463.3	559.5	992.6	739.7
S1pr4	-0.2305	0.4259	4266	6253	4795	4201	4644	4770	5729	5039
Ccr5	0.2376	0.5176	1961	3489	5491	14160	13110	11820	7303	14920
Ccr2	0.5095	0.1311	2929	6018	5340	6965	8060	4927	3874	5554
Cx3cr1	1,282	0.0001259	773	512.3	1343	11230	8912	8338	3213	4659
S1pr5	1,914	1,22E-05	13970	17300	15730	14670	14940	18520	4554	4049
Ccl4	1,925	1,25E-02	118900	33200	24910	34920	33670	65010	13300	10390
Ccl3	2,108	3,83E-04	46190	15510	13140	12380	12820	22930	4887	2654
Ccl9	2,21	1,81E-03	1118	581.9	471	857.9	1109	1311	219.4	256.6
Ccl5	2,762	9,42E-05	593800	146300	95150	167700	185900	255700	37360	23300

Fc receptors and adhesion molecules

Gene symbol	FC: NK + IL-12 / eNK	pValue	NK (1)	NK (2)	NK (3)	NK + IL-12 (1)	NK + IL-12 (2)	NK + IL-12 (3)	eNK (1)	eNK (2)
Fcgrt	-2,279	8,06E-05	711,4	445,4	1645	59,83	151,2	203	700,1	651,9
Cd7	-1,891	3,78E-04	11090	5438	5081	6188	8185	11820	38400	27110
Fcgr3	-1,169	0,001722	3195	1271	1723	1026	857,5	1105	2917	1628
Cd2	-1,134	0.003467	48110	15950	13690	9556	14590	14080	38400	18390
Cd69	-1,131	0,00207	1764	1940	1850	5742	5504	10810	13590	18790
Cd27	-0.9547	0.008032	1379	1268	948.9	994.8	1409	986.3	2518	1920
Fcgr2b	-0,6953	0,07681	1524	1949	1627	837,6	830,5	778,4	1661	1018
Itga1	-0.6115	0.2027	291.4	123.5	3500	1316	1177	532.8	931	2172
Itgb1	-0.2722	0.5192	5224	9952	17380	13100	13630	5563	9271	16940
Cd38	0.07408	0.8391	1260	1602	2932	4998	6883	3193	3945	5691
Cd9	0.08806	0.7794	12500	6827	9416	4418	5210	6042	5994	3960
Cd84	0.1322	0.6522	4828	4273	3886	5137	5510	5238	4502	5250
Itgax	0.2318	0.4956	474.7	816.8	598.5	3344	3379	2281	2156	3003
Cd53	0.2447	0.5199	14670	15160	16570	27650	32420	37600	33670	21990
Icam1	0.4489	0.2147	3394	5657	7688	16630	16200	24810	11970	16370
Itgal	0.6143	0.1563	20170	28160	30540	35240	35300	19900	13380	26300
Itgb2	0.9716	0.02894	25800	53930	50660	111500	131600	115900	75400	48340
Itga2	1,021	0.005444	1822	1840	2475	2741	3986	2133	1430	1514
Fcer1g	1,806	5,13E-06	142900	32980	21660	17550	22620	25280	8371	4289
Itgam	2,013	1,66E-03	4542	7965	8592	17560	15980	7601	2841	4025

Activating and Inhibitory receptors

Gene symbol	FC: NK + IL-12 / eNK	pValue	NK (1)	NK (2)	NK (3)	NK + IL-12 (1)	NK + IL-12 (2)	NK + IL-12 (3)	eNK (1)	eNK (2)
Cd160	-1,918	9,50E-06	1501	633.2	513.2	1515	1639	2698	8595	6314
Cd226	-1,531	3,89E-03	1323	1581	1888	3555	3121	2201	6863	10390
Klrb1f	-0.5571	0.0779	5332	5664	4655	4720	4371	6863	8951	6869
Klrc2	-0.2126	0.5613	737.4	948.1	973.9	1185	1565	1854	2119	1481
Klrc1	-0.1763	0.5902	3032	3906	4203	4844	6008	7791	8576	5641
Itgb7	-0.1405	0.6755	3022	9920	10090	14790	16170	18510	16140	20510
Klrc3	-0.1307	0.7009	1126	1735	1841	1911	2373	2814	3213	2036
Cd48	0.0205	0.952	7154	3296	5390	11590	13440	14610	16580	9835
Cd96	0.1672	0.6785	889.3	1743	2402	831.5	927.7	1029	959.2	716.5
Klrg1	0.321	0.4294	15990	10850	7539	30810	36070	47010	39320	22280
Klrk1	0.3596	0.2985	17010	18400	17110	20830	22760	25600	20050	16310
Klri1	0.64	0.06052	1516	2346	1816	2371	2628	2493	1944	1304
Klrd1	0.6686	0.03603	26600	14350	7139	7867	8328	9221	6615	4188
Cd244	0.6869	0.03666	8054	10770	8668	15150	15020	15030	11250	7719
Ncr1	0.7607	0.08951	104500	93090	83630	125300	138900	161000	98420	70900
Klri2	0.9705	0.001978	5816	12490	12230	13450	13660	13170	7269	6590
Klra7	1,599	7.2e-06	7332	4396	3375	5357	6330	6836	2744	1392
Klre1	1,901	3,46E-06	16530	8475	5592	6250	6252	7080	2066	1473
Klra9	2,165	1,20E-02	2259	1012	664.8	800.1	1008	1180	295.7	155
Klra3	2,243	1,35E-06	16900	8515	6672	8499	9948	12210	2843	1535
Klrb1c	2,309	5,40E-09	14040	14780	12340	7259	8816	10630	1992	1641
Klra1	2,947	1,34E-07	1536	810.7	641.5	1146	1253	1497	175.5	164.5
klra4	4,068	3,85E-07	276,6	130,4	394,6	217,4	226,5	225,7	18,19	0
klra6	2,736	0,00122	98,77	89,96	217,5	179,2	157,8	129,7	36,38	0

Effector molecules

Gene symbol	FC: NK + IL-12 / eNK	pValue	NK (1)	NK (2)	NK (3)	NK + IL-12 (1)	NK + IL-12 (2)	NK + IL-12 (3)	eNK (1)	eNK (2)
Tnfsf10	-1,669	0.005336	128.6	100	576.9	489.8	565.9	99.03	948.7	1525
Gzmk	-0,4332	0.2772	451.5	236.6	350.5	896.4	816.5	973.5	1547	905.1
Fasl	0.7925	0.02776	13950	18320	20990	25880	24630	33750	16320	16420
Gzma	1,188	0.02575	862400	238600	160300	285200	341200	417300	209900	1,00E+05
Prf1	2,375	7,91E-04	29410	71040	71320	167700	200200	200400	38170	36590
Gzmb	4,607	8,47E-23	33290	26010	17940	25970	26120	35660	1457	973.1

Transcription factors

Gene symbol	FC: NK + IL-12 / eNK	pValue	NK (1)	NK (2)	NK (3)	NK + IL-12 (1)	NK + IL-12 (2)	NK + IL-12 (3)	eNK (1)	eNK (2)
Rora	-2,22	9,18E-06	262.7	760.2	684.6	753.4	612.4	1180	3018	4932
Nfkb1	-1,172	0.0001173	1427	2376	1509	3619	3130	4056	9771	6673
Arntl	-0.8437	0.05393	198.4	832.4	765.5	870	389.9	687.3	1154	1200
Foxo1	-0.366	0.3347	1350	681.1	425.4	962.3	894.2	1297	1563	1178
Runx1	-0.2989	0.3271	1725	2439	1660	2904	3146	2972	3181	4280
Nfatc3	-0.2436	0.487	1560	1851	1510	1487	1414	1303	1885	1475
Ets1	-0.1468	0.6728	4511	10350	8965	9253	8517	5303	6916	10270
Tox	-0.1394	0.6493	602	1371	1085	2459	3042	3161	3279	3148
Klf13	-0.1191	0.696	10950	10890	8026	5754	7534	6701	8421	6236
Foxn2	-0.07505	0.8843	736.1	999.5	1744	1331	1834	570.4	755.4	1885
Gata3	0.0728	0.8259	2750	4504	3530	3447	3806	3915	4690	2494
Eomes	0.1198	0.7048	6583	5350	2649	2807	2425	3391	2874	2472
Foxp1	0.1463	0.6317	2498	2743	3671	3232	3012	2823	2820	2702
Stat1	0.1647	0.6518	1684	7177	7653	18780	23840	14160	14850	19250
Klf2	0.1756	0.6414	9142	3091	1141	1477	1475	1747	1806	1008
Tcf3	0.1914	0.6762	1142	1287	694.9	812.2	727.9	523.9	771.1	453
Nfkb2	0.2648	0.5076	439.2	1455	1293	2478	1474	1622	1117	1997
Id2	0.2874	0.4755	36560	28700	16880	45640	45550	61960	50120	34560
Stat4	0.3696	0.2303	4985	11040	11530	6043	8724	7627	5704	5967
Tbx21	0.6087	0.1093	9132	27230	25870	37200	46050	46610	30730	26680
Nfil3	1,452	0.009795	1060	1275	712.1	1353	604.8	1441	206.9	621.8
